# Encoding of goal-relevant stimuli is strengthened by emotional arousal in memory

**DOI:** 10.3389/fpsyg.2015.01173

**Published:** 2015-08-10

**Authors:** Tae-Ho Lee, Steven G. Greening, Mara Mather

**Affiliations:** ^1^Department of Psychology, University of Southern California, Los Angeles, CA, USA; ^2^Davis School of Gerontology, University of Southern California, Los Angeles, CA, USA; ^3^Neuroscience Graduate Programs, University of Southern California, Los Angeles, CA, USA

**Keywords:** emotion, arousal, memory, top-down goal, fear-conditioning, arousal-biased competition, emotion-induced memory enhancement

## Abstract

Emotional information receives preferential processing, which facilitates adaptive strategies for survival. However, the presence of emotional stimuli and the arousal they induce also influence how surrounding non-emotional information is processed in memory (Mather and Sutherland, 2011). For example, seeing a highly emotional scene often leads to forgetting of what was seen right beforehand, but sometimes instead enhances memory for the preceding information. In two studies, we examined how emotional arousal affects short-term memory retention for goal-relevant information that was just seen. In Study 1, participants were asked to remember neutral objects in spatially-cued locations (i.e., goal-relevant objects determined by specific location), while ignoring objects in uncued locations. After each set of objects were shown, arousal was manipulated by playing a previously fear-conditioned tone (i.e., CS+) or a neutral tone that had not been paired with shock (CS–). In Study 1, memory for the goal-relevant neutral objects from arousing trials was enhanced compared to those from the non-arousing trials. This result suggests that emotional arousal helps to increase the impact of top-down priority (i.e., goal-relevancy) on memory encoding. Study 2 supports this conclusion by demonstrating that when the goal was to remember all objects regardless of the spatial cue, emotional arousal induced memory enhancement in a more global manner for all objects. In sum, the two studies show that the ability of arousal to enhance memory for previously encoded items depends on the goal relevance initially assigned to those items.

## Introduction

Events or stimuli charged with emotional meaning stand out. For example, emotional faces, emotional words and emotional scenes are more likely to be detected (e.g., Amting et al., 2010) and remembered (e.g., Mather and Nesmith, 2008) than neutral ones. This preferential processing of emotional information is considered an adaptive strategy for survival and well-being (Öhman and Mineka, 2001), as attending to a threatening stimulus such as a snake or spider can help one avoid harm. In addition, the presence of such threatening stimuli, and the emotional arousal they induce, can also influence how surrounding non-emotional information is processed. For example, seeing a snake on a hiking trail could also lead you to better recall a distinctive feature of that trail so as to avoid it in the future.

Recent studies have shown that emotion’s influence carries over to spatially or temporally adjacent non-emotional stimuli. For example, presenting an emotionally arousing cue (e.g., an emotional face or scene) influences visual perception of subsequent neutral items (Becker, 2009; Bocanegra and Zeelenberg, 2009; Ihssen and Keil, 2009; Wang et al., 2012; Lee et al., 2014a) and memory consolidation of preceding neutral items (Anderson et al., 2006; Dolcos and McCarthy, 2006; Knight and Mather, 2009). Yet these carryover effects of emotional arousal are not uniform. Some studies reveal emotion-induced enhancement of visual processing (e.g., Becker, 2009), whereas others reveal impairment due to emotion (e.g., Wang et al., 2012). The memory literature has also found both emotion-induced retrograde amnesia (e.g., Dolcos and McCarthy, 2006), and emotion-induced retrograde enhancement (e.g., Anderson et al., 2006).

The arousal-biased competition (ABC) model posits that emotional arousal can both enhance and impair processing of different representations simultaneously (for reviews, see Mather and Sutherland, 2011; Mather et al., 2015). ABC builds upon biased competition models (e.g., Desimone and Duncan, 1995), in which stimuli compete for neural representation in a mutually inhibitory fashion, with the competition being biased in favor of goal-relevant, or perceptually salient, stimuli (Itti et al., 1998; Beck and Kastner, 2009). The ABC model proposes that emotional arousal increases gain, there by enhancing mental representations of high-priority items even more than typical biased competition processes while inhibiting representations of low-priority items (i.e., “winner-take-more” and “loser-take-less”). Thus, the relative discrepancy between these types of stimuli is amplified. Priority in the competition is determined by both bottom-up perceptual saliency (e.g., stimuli that move suddenly or are brighter than their surroundings; Itti et al., 1998) and top-down goal-relevancy (e.g., finding a friend in a crowd; Beck and Kastner, 2009). Consistent with ABC, recent studies have demonstrated that emotional arousal facilitates subsequent perception of non-emotional, visually salient stimuli, while impairing perception of non-salient stimuli (Lee et al., 2012, 2014b; Sutherland and Mather, 2012, 2015).

Thus, the ABC model posits that a momentary increase in arousal will influence later memory for currently active representations and that whether arousal will lead to enhancement or impairment in memory for what was encoded right beforehand will depend on the priority of that information. In a study testing this hypothesis (Sakaki et al., 2014), participants saw a sequence of objects on each trial. In each sequence, there was one scene image that looked different than the objects. This scene served as a perceptual oddball. On half of the trials the oddball image was emotionally arousing. All participants were asked to report the identity of one of the items at the end of each list, but which item they had to report differed: One group of participants was asked to report the identity of the oddball. A second group was asked to report the object that immediately preceded the oddball. So in one condition, the oddball-1 item had high priority, whereas in the other condition it was just one item in a sequence. Memory for all the oddball-1 items was tested at the end of the session. Memory for the oddball-1 items was modulated by the subsequent emotional oddball in opposite directions in the two conditions. When the oddball-1 item had high priority, subsequent emotion enhanced long-term memory for it, whereas when it had low priority, subsequent emotion impaired long-term memory for it. Importantly, the direction of these effects varied as a function of goal-directed priority within the same task structure (Sakaki et al., 2014).

In the current study, we wanted to examine whether arousal after mental representations have been activated influences the degree of competition among different stimuli or whether it simply acts differentially depending on particular items’ priority. Thus, we tested how arousal would modulate short-term memory consolidation when multiple target items were shown together. In particular, we were interested in whether multiple prioritized items could all benefit from subsequent arousal, and whether the amount of enhancement would depend on the degree of prioritization. To create top-down priority during the study, a spatial-attention cueing paradigm was adapted to a memory-encoding task in which participants tried to remember the neutral objects presented in the cued locations. We used a fear-conditioned tone (i.e., CS+) to manipulate participant’s arousal levels on a trial-by-trial basis during the study. In Study 1, ABC predicted an arousal by task-relevance interaction such that post-encoding arousal would enhance memory for the objects in the cued locations (i.e., goal-relevant objects) more than for the objects in the uncued locations (i.e., non-relevant objects). In Study 2, we asked participants to prioritize all objects regardless of the cue location, but to give the cued objects highest priority. Thus, a key question in Study 2 was what would happen for the items that had some—but not the most—priority under arousal, and whether having matching items in this condition would matter.

## Study 1

### Materials and Methods

#### Participants

Based on previous studies examining the impact of fear-conditioning on behavior in which the number of participants varied between 25 and 40 participants (Lee et al., 2009a,b, 2014a), we included 33 participants (nine male; *M*_*age*_ = 20.22, range 18–27) with corrected-to-normal vision volunteered for this study and gave informed consent in accordance with University of Southern California Institutional Review Board guidelines.

#### Stimuli and Apparatus

We used 240 color photographs (5.5 × 5.5°) of real-world objects (e.g., fruit, car, animal, tools, etc.) from a previously published set of object stimuli (http://cvcl.mit.edu/mm/objectCategories.html). Electric shock administered with a human shock stimulator (Coulbourn Instruments, Allentown, PA, USA) served as an unconditioned stimulus (US) during the fear-conditioning session. The intensity of the shock was individually set by each participant at a level that was “*unpleasant but not painful*” (total *M*_*intensity*_ = 1.81 mA, total range 1.0–4.0 mA). Two tones (500 and 1400 Hz) were adopted as conditioned stimuli (i.e., CSs).

Skin conductance response (SCR) was recorded at 1,000 Hz sampling rates with the MP-150 system (BIOPAC, Goleta, CA, USA). The SCR was calculated by subtracting a baseline (average signal between 0 and 1 s) from the maximum peak amplitude during the 1–7 s time window following the CS onset (Lee et al., 2014a,b). The trials that included shocks were excluded in subsequent analyses.

All procedures were performed in a dimly lit soundproof room at a viewing distance of 57 cm from a 19-in. CRT monitor (85 Hz refresh ratio; 1280 × 960 resolution).

#### Procedure

In an initial fear-conditioning phase, one tone was paired with electric shock (CS+) while the other tone was not paired with shock (CS–). To avoid ambiguity participants were told which tone signaled shock, and the tone-shock pairings were counterbalanced across participants (14 participants were conditioned with the high-pitch tone as the CS+). The US was delivered to the third and fourth fingers of the left hand. Each trial began with a fixation-cross jittered to appear for 7 to 10 s (i.e., inter-trial interval; ITI). Then one of the CS tones played for 1 s. On CS+ trials, a shock was delivered for 0.5 s after a 1.5 s-inter stimulus interval (ISI). We adopted a trace-conditioning paradigm to maintain participant’s arousal level (or anticipation for the US) even after each CS tone terminated. In order to ensure that participants attended to the tones, they were asked to indicate the type of tone (i.e., low- or high-pitched) with a button press. To slow extinction learning for CSs, a partial reinforcement schedule (50%) was used; a total of 30 trials were presented in a random order: 10 CS+ with shock; 10 CS+ without shock; 10 CS– tones. Participants were not informed about the probability of the US delivery. To confirm the success of the conditioning, SCR was measured during the conditioning phase.

**FIGURE 1:**
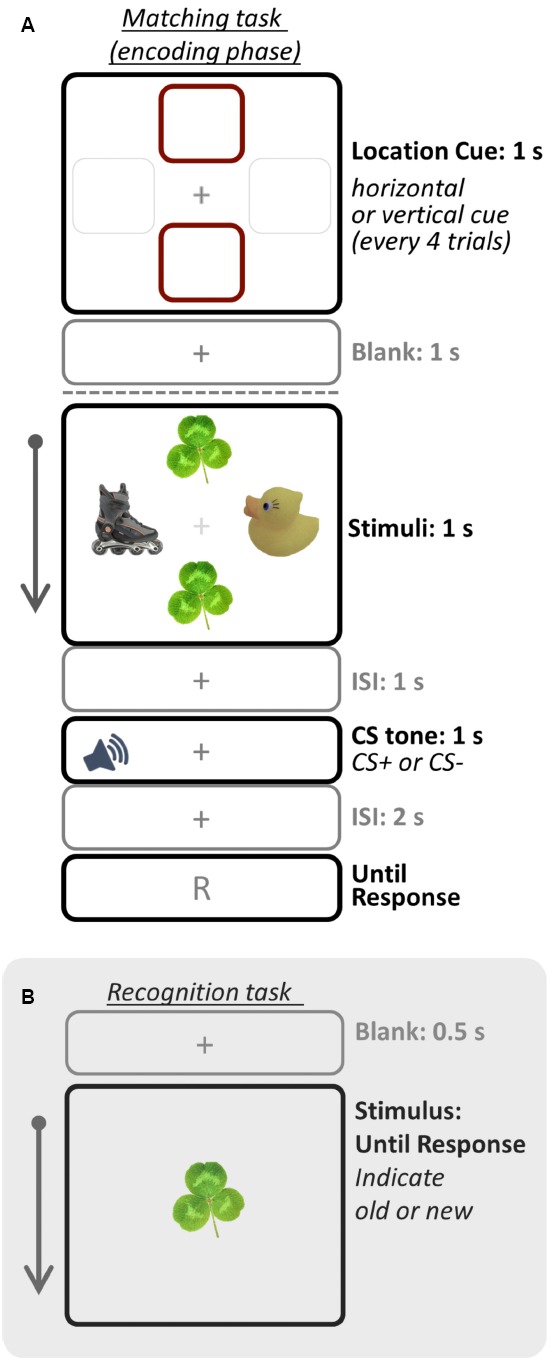
**Schematic illustration of one trial for the (A) matching task (encoding phase) and (B) recognition task (test phase).** In Study 1, participants were asked to remember only objects in the cued location whereas in Study 2 they were asked to remember all objects. Therefore, only objects in the cued location were top-down prioritized in Study 1, but all objects were prioritized in Study 2. The recognition task was administered right after the matching task session. Images were drawn not to scale.

Following the conditioning phase, the encoding phase was administered in which participants were asked to remember objects in the cued location (i.e., prioritized objects) while ignoring the other objects (Figure 1A). Once every four trials, participants were given a 1-s cue to indicate the location (either vertical or horizontal; 6.0°eccentricity) to be attended for the next four trials. After the location cue, trials began with the object display for 1 s, followed by a 1-s blank screen. Then either the CS+ or CS– tone played for 1 s while a fixation cross was shown, followed by a 2-s blank screen. In order to ensure that participants attended to the task, they were also asked to indicate whether a pair of objects in the cued location was the same or different (i.e., matching task). A 5-s ITI was presented between trials. There were two runs for the encoding phase (16 CS+ and 16 CS– trials per run), and each run was repeated twice across the study. To minimize extinction of conditioned responses, three additional CS+ trials with shock were presented randomly in each run (i.e., booster trials).

Finally to test the primary hypothesis, an immediate recognition memory task with all object images used in the encoding phase (i.e., old items) and additional 48 objects as lures (i.e., new items) was administered after the matching task (Figure 1B). On each trial, participants were presented with one object item, and asked to indicate whether the item was “old” or “new” by pressing a key in a self-paced manner.

### Results and Discussion

**FIGURE 2:**
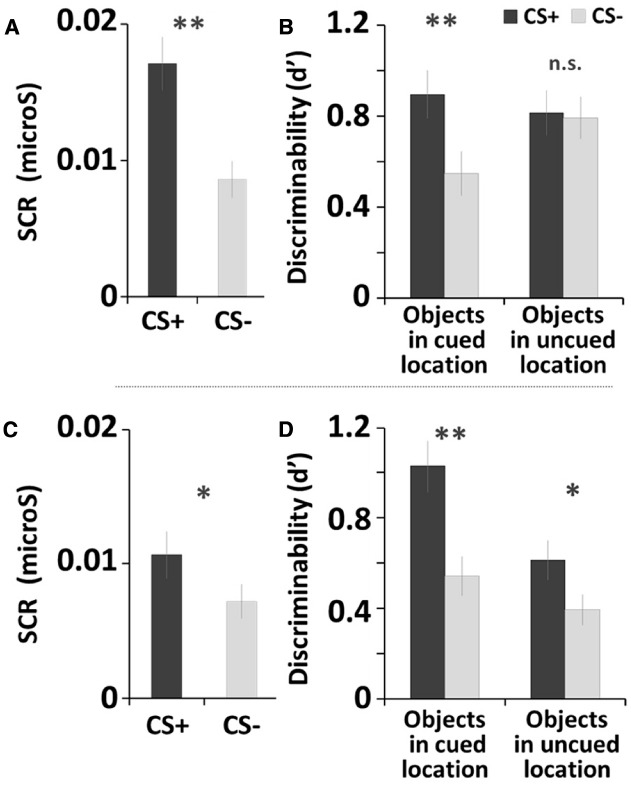
**Fear conditioning and memory recognition task results of Study 1 (A,B) and Study 2 (C,D).** Error bars represents standard errors. **p < 0.001; *p < 0.05.

For the fear-conditioning session, the SCR results confirmed that fear conditioning successfully manipulated arousal in the current study, as the CS+ yielded greater SCR than did the CS– tone, *t*(32) = 6.41, *p* < 0.001, Cohen’s *d* = 2.70 (Figure 2A).

For the memory task, we used individual *d*-prime (*d*′) scores to obtain a quantitative measure of memory accuracy, as *d*′ indicates how accurately participants discriminates between signal (old items) and noise (new items) (Macmillan and Creelman, 2004). The *d*′ score was calculated by taking the difference between z-scored proportions of hits [*p*(H): correct responses to old items] and false alarms [*p*(FA): incorrect responses to new items]; *p*(H) and *p*(FA) were adjusted as follows: *p*(H) = 1 was recalculated as 1–1/(2N), *p*(FA) = 0 was recalculated as 1/(2N), where N is the maximum number of hits or false alarms possible. Higher *d*′ scores indicate greater memory accuracy. A 2 (*Arousal Condition*: CS+, CS–) × 2 (*Stimulus Type*: objects in cued locations, objects in uncued locations) repeated-measures ANOVA was conducted on the d-prime score^1^. There were main effects of *Arousal Condition*, *F*(1, 32) = 22.46, *p* < 0.001, ${\text{η}}_{p}^{2}$ = 0.41, showing that overall memory accuracy was higher for objects with the CS+ tone (*M*_*CS*+_ = 0.86) than with the CS– tone (*M*_*CS*–_ = 0.67). There was also a main effect of *Stimulus Type*, *F*(1, 32) = 4.72, *p* < 0.05, ${\text{η}}_{p}^{2}$ = 0.13, showing that participants remembered objects in the uncued locations better (*M*_*cued*_ = 0.72) than objects in the cued locations (*M*_*uncued*_ = 0.80) regardless of arousal condition. Finally, a significant *Arousal Condition* × *Stimulus Type* interaction was observed, *F*(1, 32) = 30.55, *p* < 0.001, ${\text{η}}_{p}^{2}$ = 0.49, as the differences between CS+ and CS– tones was only seen for items shown in the cued locations. Subsequent pairwise comparisons (least significant difference; *LSD*) showed that the post-encoding CS+ tone enhanced later recognition of objects shown in the cued locations, compared with the CS– tone (*p* < 0.001; Figure 2B; see also Table 1). In contrast, there was no significant difference between CS+ and CS– tones for objects in the uncued locations (i.e., non-prioritized stimuli). We also compared memory accuracy within the same arousal condition, and found that there was a marginally significant difference in d-prime for objects in the cued location compared to the uncued location in the CS+ trials (*p* = 0.076).

**TABLE 1:** **Averaged recognition accuracy (standard error) in both Study 1 and Study 2**.

**Stimulus condition**		**Arousal condition**	**Study 1 (*N* = 33)**		**Study 2 (*N* = 27)**	
			***Hit***	***d*′**	***Hit***	***d*′**
Old	Object in cued location	CS+	0.276 (0.022)	0.895 (0.105)	0.421 (0.031)	1.028 (0.115)
		CS–	0.183 (0.020)	0.547 (0.098)	0.284 (0.027)	0.612 (0.087)
	Object in uncued location	CS+	0.246 (0.016)	0.814 (0.099)	0.268 (0.031)	0.542 (0.088)
		CS–	0.242 (0.019)	0.792 (0.093)	0.218 (0.023)	0.393 (0.067)
New	New object		*False alarm* .092 (.018)		*False alarm* 0.139 (0.022)	

In sum, as expected, the effects of arousal were most apparent for cued items that had top-down high priority. However, arousal had no significant impairment effect on uncued items, suggesting that post-perception arousal does not amplify competition between multiple items. Study 2 was designed to test whether arousal only enhances retrograde memory when there was one high priority item (the cued matching condition) or whether multiple prioritized items can benefit from arousal, and also to see if the finding that effects of arousal were strongest in the matching condition would replicate.

## Study 2

Study 1 suggests that arousal amplifies existing effects of top-down priority, but we did not see impairment of competing, lower priority stimuli under arousal, as would be predicted from a competition model. An alternative model is that arousal can enhance multiple high priority items simultaneously. To test this, we needed to increase the priority of the non-cued items. Thus, in Study 2, participants were told that they should remember all objects on the screen, but only two of them were also cued. Thus, Study 2 tested whether arousal only enhances short-term memory consolidation of the highest priority item or whether it can enhance it for multiple high-priority items. Objects in the cued location were still the focus of the matching task as in Study 1. All experimental method and materials for Study 2 were the same as for Study 1, except for the following: (1) There were 27 participants (11 male; *M*_*age*_ = 20.30; range 18–29; 18 were conditioned with the high-pitched tone); (2) The mean shock intensity was = 1.41 mA, range 0.6–4.0 mA; (3) The task goal (i.e., priority) during the encoding phase was changed such that participants were asked to remember all objects regardless of the cued location for matching.

Thus, in Study 2, all items were prioritized for the memory task, but cueing two items gave them higher priority than uncued items. If experiencing arousal after perceiving multiple visual objects amplifies competition among the representations of these objects, arousal should enhance memory for the highest priority objects but impair memory for lower priority competing objects. In contrast, if arousal generally enhances anything that has high priority (i.e., is highly activated) at the moment arousal is experienced, then if all the objects have high enough priority, none should be inhibited.

### Results and Discussion

The SCR analysis confirmed the success of the fear conditioning, as the CS+ yielded significantly greater SCR than the CS–, *t*(26) = 2.58, *p* < 0.05, *d* = 1.13 (Figure 2C).

For memory accuracy, a repeated-measures ANOVA^2^ revealed a significant main effect of *Arousal Condition*, *F*(1, 26) = 27.29, *p* < 0.001, ${\text{η}}_{p}^{2}$ = 0.51, as memory was better with the CS+ tone (*M*_*CS*+_ = 0.79) than with the CS– tone (*M*_*CS*–_ = 0.50). There also was a main effect of *Stimulus Type*, *F*(1, 26) = 12.24, *p* < 0.005, ${\text{η}}_{p}^{2}$ = 0.32; as shown in Table 1, memory was better for items in the cued location (*M*_*cued*+_ = 0.82) than in the uncued lovation (*M*_*uncued*_ = 0.47). Subsequent pairwise comparisons (*LSD*) showed that the CS+ increased recognition for objects in both the cued location (*p* < 0.001) and the uncued location (*p* < 0.05; Figure 2D), but that the arousal-induced memory advantage was greater in the cued location, leading to a significant *Arousal Condition* × *Stimulus Type* interaction, *F*(1,26) = 7.29, *p* < 0.05, ${\text{η}}_{p}^{2}$ = 0.22. In sum, overall the CS+ enhanced later recognition of prioritized objects compared to the CS–, but for the objects in the cued location most.

## General Discussion

In the current study, we examined whether arousal enhances information processing only for the highest priority information at a particular moment, or whether it can enhance priority for other prioritized items. The ABC model predicts that emotional arousal selectively enhances information processing for stimuli that are perceptually conspicuous or relevant to task goals (Mather and Sutherland, 2011) while impairing lower priority stimuli, but previous studies have not tested the role of competition in this process.

In the current two studies, participants were shown a set of objects and cued to two of them, and then they were exposed to an emotionally arousing stimulus (i.e., CS+) after encoding (Figure 1A). In Study 1, whereas memory for goal-relevant matching objects was enhanced on the CS+ compared to the CS- trials, no enhancement was observed for uncued, non-priority, objects. This suggests that the strength of top-down goal priority was amplified by emotional arousal, but competing items were not suppressed. In Study 2, emotional arousal produced memory enhancement for objects in both cued and uncued locations of the matching task when the task goal was to remember all objects irrespective of the cued location. Furthermore, arousal enhanced memory for objects in the cued location to a greater extent than it did for objects in the uncued locations. Taken together, these findings are consistent with the notion that arousal modulates representations based on their priority, amplifying the effect of top-down goals on memory consolidation, but that these modulation effects do not depend on competitive interactions among representations.

This is the first study to show that arousal induced after seeing a simultaneous display of multiple items enhances memory for the highest priority items. Previous findings of enhanced retrograde memory only had one preceding item shown and tested (Knight and Mather, 2009; Sakaki et al., 2014). The results from the current study also indicate that representations of other prioritized items (that are not necessarily the highest priority in the current set of items) can also be enhanced by subsequent arousal. Models of biased competition often suggest a “winner-take-all” mechanism (Itti et al., 1998; Beck and Kastner, 2009). Yet top-down goals sometimes require the prioritization of more than one item at a time, which would be impossible to do with a winner-take-all process.

In Study 1 we also found differential levels of enhancement based on item priority, but no strong indication of suppression of low priority items. This is contrary to the effects observed for low-priority oddball-1 items in Sakaki et al. (2014). This lack of suppression suggests that merely being encoded in the presence of higher priority items is not a sufficient basis for something to be the target of suppression under arousal. Instead, it may be that a stronger initial competition between stimuli or even an initial suppression process may be necessary. For instance, having one stimulus overlaid on top of another would induce stronger competition, which arousal should amplify (e.g., Ponzio and Mather, 2014). Another possibility is that a process akin to inhibition of return (IOR) produced greater encoding of items in the uncued location (e.g., Posner et al., 1985). Future studies are needed to determine if the degree of initial competition in visual processing or attentional focus in relation to the IOR effects can explain the current study’s lack of memory suppression of low priority objects during emotional arousal (e.g., Lee et al., 2012).

One limitation of this study is that we only used negative stimuli to increase the participants’ arousal levels as negative stimuli generally induce stronger arousal responses than positive stimuli (Lang et al., 1998; Baumeister et al., 2001). This means that we cannot be sure whether our results are due to the effects of negatively valenced emotional arousal *per se* or emotional arousal more generally. There are two additional possibilities to consider, both of which might influence the breadth of attention. First, that a safety feeling was produced upon hearing a CS– tone. Second, during the CS+ condition participants might have engaged in regulatory processes to avoid processing the CS+ tone. By influencing the breadth of attention, either of these two processes could change the initial encoding of the viewed images. Indeed previous research has shown that positive and negative stimuli have differential effects on the breadth of attention selectivity (e.g., Schmitz et al., 2009) and encoding processes (e.g., Kensinger and Corkin, 2004). However, a more recent study revealed that emotional arousal induced by either positive or negative stimuli enhances memory consolidation for previously viewed objects (Sakaki et al., 2014). Specifically, arousing stimuli enhanced later memory of preceding goal-relevant objects while impairing later memory of preceding goal-irrelevant ones. Thus, although in certain contexts positive and negative valences may have differential effects on memory, more recent evidence suggests that arousal amplifies the gain on strong vs. weak mental representations, regardless of whether that arousal was induced via positive or negative stimuli.

An important direction for future research is to better understand the neural bases of these retrograde effects of arousal that vary depending on the priority of each specific stimulus representation. A recent proposal is that, during moments of phasic arousal such as those induced by a CS+ tone, interactions between the brain’s primary excitatory neurotransmitter, glutamate, and the concurrent release of a neuromodulator during arousal (i.e., norepinephrine) lead to local hotspots at the sites of highly active neurons, amplifying high priority representations (Mather et al., 2015). This model provides one potential explanation for the current findings. Namely, that arousal has differential effects on representations depending on their level of priority.

Taken together our studies demonstrate the ability of fear-induced arousal to enhance memory for previously encoded items and to do so differentially depending on the level of initial priority. This process operates on a moment-by-moment basis, with brief fluctuations in arousal (such as those induced by a conditioned tone) modulating currently activated representations such that higher priority representations are later remembered even better than they would have been otherwise. This targeted retrograde enhancement is likely one way that our arousal system helps us remember the things that really matter.

### Conflict of Interest Statement

The authors declare that the research was conducted in the absence of any commercial or financial relationships that could be construed as a potential conflict of interest.

## Footnotes

1 Repeating this analysis on the hit rate for old items revealed the same patterns. There was a significant *Arousal Condition* × *Stimulus Type* interaction, *F*(1, 32) = 25.17, *p* < 0.001, ${\text{η}}_{p}^{2}$ = 0.44, indicating that the CS+ led to higher recognition for objects in the cued-location than did the CS– tone. There was also a main effect of *Arousal Condition*, *F*(1, 32) = 21.47, *p* < 0.001, ${\text{η}}_{p}^{2}$ = 0.40, as memory was higher for objects with the CS+ tone than for objects with the CS– tone (*M*_*CS*+_ = 0.26 vs. *M*_*CS*–_ = 0.21). Note that the hit rate data was not z-scored for this analysis.

2 For hit rates, the same patterns were observed: a significant *Arousal Condition* × *Stimulus Type* interaction, *F*(1, 26) = 8.33, *p* < 0.01, ${\text{η}}_{p}^{2}$ = 0.24, significant a main effect of *Stimulus Type*, *F*(1, 26) = 13.97, *p* < 0.001, ${\text{η}}_{p}^{2}$ = 0.35 (*M*_*cued*_ = 0.35 vs. *M*_*uncued*_ = 0.24) and *Arousal Condition*, *F*(1, 26) = 32.55, *p* < 0.001, ${\text{η}}_{p}^{2}$0= 0.56 (*M*_*CS*+_ = 0.35 vs. *M*_*CS*–_ = 0.25). Note that the hit rate data was not z-scored for this analysis.
